# A DNA Data Storage Method Using Spatial Encoding Based Lossless Compression

**DOI:** 10.3390/e26121116

**Published:** 2024-12-20

**Authors:** Esra Şatır

**Affiliations:** Computer Engineering Department, Düzce University, 81620 Düzce, Turkey; esrasatir@duzce.edu.tr

**Keywords:** DNA storage, DNA data hiding, DNA encoding, DNA compression, lossless compression

## Abstract

With the rapid increase in global data and rapid development of information technology, DNA sequences have been collected and manipulated on computers. This has yielded a new and attractive field of bioinformatics, DNA storage, where DNA has been considered as a great potential storage medium. It is known that one gram of DNA can store 215 GB of data, and the data stored in the DNA can be preserved for tens of thousands of years. In this study, a lossless and reversible DNA data storage method was proposed. The proposed approach employs a vector representation of each DNA base in a two-dimensional (2D) spatial domain for both encoding and decoding. The structure of the proposed method is reversible, rendering the decompression procedure possible. Experiments were performed to investigate the capacity, compression ratio, stability, and reliability. The obtained results show that the proposed method is much more efficient in terms of capacity than other known algorithms in the literature.

## 1. Introduction

Information technology has undergone extreme development in recent years, yielding new ways of processing data with secure and rapid characteristics. Inspired by the DNA (Deoxyribonucleic Acid) structure, in the near future, it will be possible to replace traditional silicon-based facilities with biological tools where computing with more than 0 and 1 is possible [1,2]. Data storage in DNA has rapidly gained interest because of its high density, long-term durability, and low maintenance costs [3]. Over time, a large amount of information that requires long-term storage has been produced by humanity. Stone, clay, papyrus, wood, paper, and other materials are reliable media in which information was stored in the late 1920s. Since then, other carriers such as silicon have been invented, and accordingly, the current digital revolution has been raised [4]. However, advances in DNA sequencing technology have reduced the cost of genome sequencing. This has led to several revolutionary advances in the genetic industry. NGS (next-generation sequencing) allows for the parallel sequencing of significant amounts of DNA and minimizes the need for comparatively inefficient fragment-cloning methods that are usually used in Sanger sequencing technologies. However, the management of such large volumes of data is an important issue, and this issue is still of interest to researchers [5].

Currently, in the Big Data era, a huge amount of data is produced every day. The global data volume is predicted to reach 175 ZB by 2025. Researchers are concerned that this increase in data volume will exceed the existing storage capacity [6]. This is because the amount of data increases by nearly 50% annually. It is predicted that this value will exceed three yottabytes by 2040. For this reason, more than 109 kg of highly pure silicon is required for their storage [7]. However, only 107–108 kg of silicon can be produced by 2040 [4]. DNA is a promising storage medium owing to its durability and ultrahigh density. It is known that one gram of DNA can store 215 GB of data, and the data stored in the DNA can be preserved for thousands of years [6]. To provide a concrete picture of the aforementioned information, the main digital information carriers are listed in Table 1. Here, it is clear that using DNA as a carrier has the main advantages in terms of capacity and lifetime, where access time is another issue to be tackled. Therefore, it is advisable to store only archival information in this biopolymer.

In the literature, there are two main approaches to DNA data storage: in vivo and in vitro. In vivo approaches are based on recombinant microorganisms that carry artificially embedded non-biological information within their genomes. Here, the information is stored via transmission from generation to generation. The main disadvantage of this approach is that only a small amount of information can be saved for living organisms [4].

Long-term in vitro storage is a widely studied area because it is suitable for computer-aided systems. This process consists of the following stages of DNA data storage: the conversion of information into nucleotide sequences (binarization), translation of binary code into DNA code (encoding), synthesis of oligos (DNA synthesis), storage of oligos under stable conditions (DNA storage after synthesis before sequencing), obtainment of a sufficient amount of informative DNA by amplification to retrieve the nucleobase code (DNA sequencing), and recovery of digital information from nucleotide sequences (decoding) [8]. Long-term in vitro storage constitutes a suitable background for researchers to reproduce novel methods by enhancing the given substages. Accordingly, this branch of research was addressed in this study.

In 2014, Hafeez et al. proposed a robust data-hiding model for watermarking DNA sequences called “DNA-LCEB”. In this model, the phenomenon of silent mutations (i.e., synonymous substitutions) for storing data in the degenerative codons of the DNA coding region has been exploited. The authors claimed that DNA-LCEB exploited the entire coding region for watermark storage, thus leading to a better storage capacity [9]. In 2018, Lee et al. proposed a method in which they addressed two approaches to reversible DNA data hiding using multiple difference expansions. In this method, the string sequences of four characters (A, T, C, G) of noncoding DNA sequences were converted into decimal-coded values, and the watermark was embedded into a coded value sequence using two approaches: DE-based multiple-bit embedding (DE-MBE) using pairs of neighboring values and consecutive DE-MBE (CDE-MBE) using previously embedded coded values [10]. In 2019, Rahman et al. presented a lossless DNA sequence hiding method to authenticate DNA sequences in the context of mobile cloud-based healthcare systems. In the proposed method, authentication data are hidden and extracted, and the DNA sequence is reconstructed without any loss of information. Security analysis and experiments regarding performance were performed in the scope of their study [11]. In 2022, Song et al. proposed a de novo strand assembly algorithm (DBGPS) using a de Bruijn graph and a greedy path search. They claimed that DBGPS had substantial advantages in handling DNA breaks, rearrangements, and indels. In the proposed study, the robustness of DBGPS was demonstrated through accelerated aging, multiple independent data retrievals, deep error-prone PCR, and large-scale simulations [3]. In 2023, Lenz and Zeh proposed a communication system in which information is transferred over many sequences in parallel. In this system, the receiver cannot control access to these sequences and can only draw from these sequences, unaware of which sequence has been drawn. Furthermore, the drawn sequences were susceptible to errors. Moreover, the information capacity was computed for a wide range of parameters and a general class of drawing distributions [12]. In 2024, Hong et al. proposed a novel Video Segmentation and Storage in DNA (VSD) method that relies on an innovative video segmentation strategy and a quadratic coding model and uses efficient indexing. This proposed encoding model based on the RS error-correcting code efficiently balances the storage density, combinatorial bio-constraints, and time efficiency, thereby reducing overhead costs [8]. Preuss et al. presented a novel approach for encoding information in DNA, using combinatorial encoding and shortmer DNA synthesis, leading to an efficient sequence design and improved DNA synthesis and readout interpretation. This method leverages the advantages of combinatorial encoding schemes while relying on existing DNA chemical synthesis methods with some modifications. They mentioned that the use of short DNA synthesis also minimized the effects of synthesis and sequencing errors [13]. Cao et al. proposed parity encoding and a local mean iteration (PELMI) scheme to achieve the robust DNA storage of images. They mentioned that the proposed parity-encoding scheme satisfied the common biochemical constraints of DNA sequences and undesired motif content. It addresses varying pixel weights at different positions for binary data, thus optimizing the utilization of the Reed–Solomon error correction. They claimed that PELMI achieved image reconstruction with general errors (insertion, deletion, and substitution) and enhanced DNA sequence quality [14].

In this study, A DNA data storage method using spatial encoding-based lossless compression was proposed. The proposed approach employs a vector representation of each DNA base in a two-dimensional (2D) spatial domain for both the encoding and decoding phases. This representation of input data renders the proposed method suitable for efficient compression. The structure of the proposed method is reversible, which renders decoding possible without any information loss. Experimental studies were performed by conducting capacity, compression ratio, stability, and reliability analyses. The obtained results show that the proposed method is much more efficient in terms of capacity than other known algorithms in the literature. Moreover, significant results were achieved in terms of the compression ratio, stability, and reliability. This paper is organized into four sections. A brief description of the DNA structure and the proposed DNA storage approach has been explained in Section 2, titled “Materials and Methods”. The performed experiments and their results are presented in Section 3, titled “Experimental Results”. Finally, a general outcome has been reached in Section 4, titled “Discussion”.

## 2. Materials and Methods

In this section, the DNA structure and details of the developed approach are explained step-by-step.

### 2.1. DNA Structure

**Figure 1:**
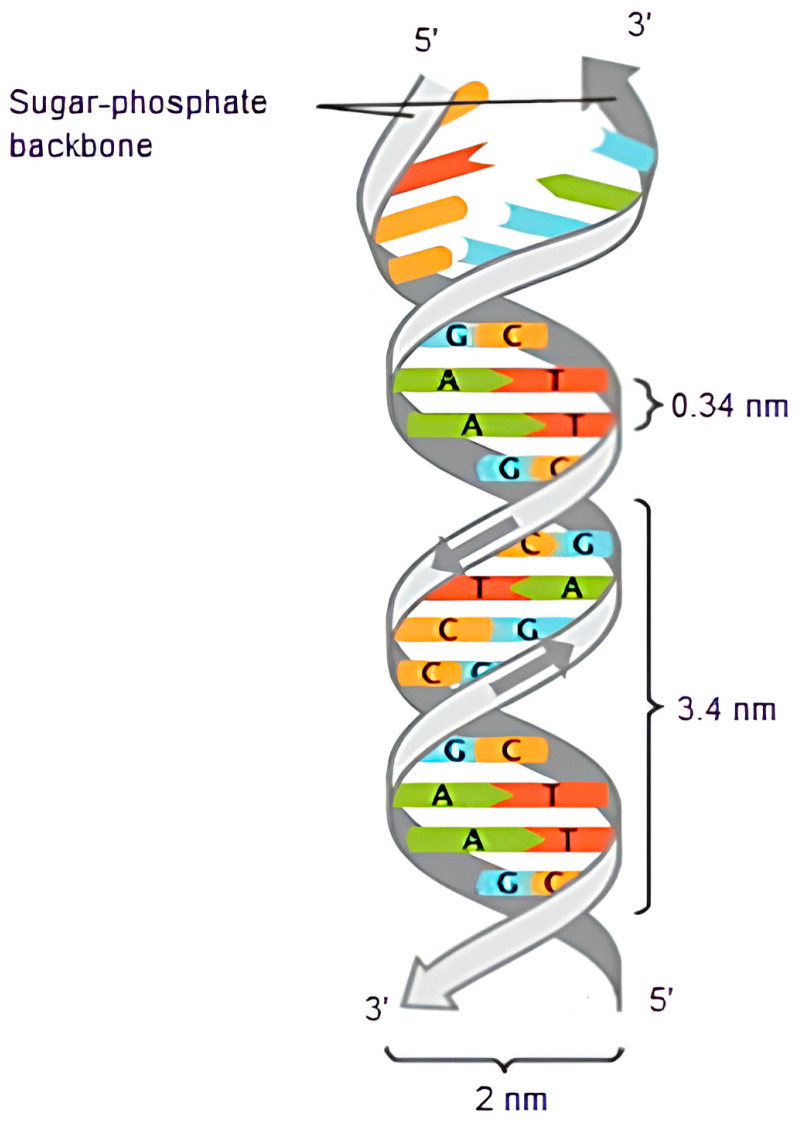
The double helix structure of DNA [15].

In the DNA structure, each nucleotide consists of one of four bases: adenine (A), guanine (G), cytosine (C), and thymine (T). A and G are purines, whereas T and C are pyrimidines. In Figure 1, the double helix structure of DNA is presented [15].

In DNA’s double-helical form, “A” joins with “T”, and “C” joins with “G”. Since DNA is known as the “instruction of life”, DNA sequences are not expected to be random and are expected to be suitable for storage. In DNA, a repeat subsequence is a copy of a previous subsequence, either forward or reverse. This yields highly repetitive patterns in the DNA sequences. When a human genetic repository is investigated, it is estimated that 55% of the human genome contains repetitive DNA nucleotides [16].

### 2.2. Encoding Phase of ASCDNA

Step 1: The binary data can be directly considered as an input for the encoding phase, or the DNA sequence that is represented in binary form via traditional 2 bits per symbol substitution (as depicted in Table 2) can be considered as an input for the encoding phase.

Let S be the original sequence, n the length of the sequence, and B the binary form of the sequence. Accordingly, B production can be formulated as follows:

(1) $$B={⋃}_{i=1}^{n}{\left(S\right)}_{2}$$

**Figure 2:**
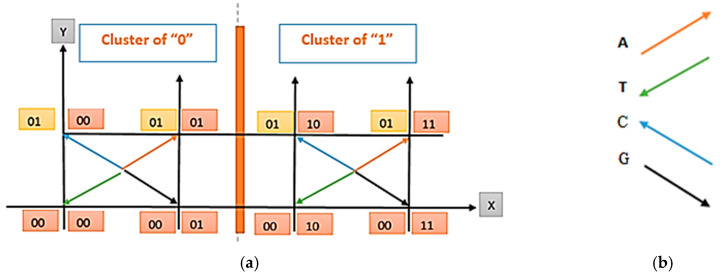
(**a**) The designed 2D spatial domain including DNA base vectors used for encoding and decoding. (**b**) Vector representation of DNA bases.

Here, we consider that base 2 represents 2 bits per symbol DNA substitution instead of algebraic base 2. The aim of employing a binary stream is to prepare an appropriate background for estimation in the 2D spatial domain, as shown in Figure 2a.

Step 2: In Step *B*, the bit stream obtained from Step 1 is divided into words called *w*. The length *w* is three. *W*, which is the set of all *ws*, can be described as follows:

(2) $$W=\{w:\forall w\in B\;;\;\left|w\right|=3\}$$

The aim of truncating the input bit stream (B) into words of length 3 (w) is to render the input suitable using the base vectors shown in Figure 2b.

Step 3: In this step, every word w of W is extended to a length of four (let us call it w′) by concatenating the MSB (Most Significant Bit) with 0. Here, the aim of concatenating the MSB of w with a 0 is first, the ease of searching and computing in the 2D spatial domain (please note that each vector has four bits in the 2D spatial domain depicted in Figure 2a). Second, the value of each w does not change by concatenating the MSB with 0 s. This operation is the first part of the compression process in the encoding phase. As a result, we obtain a new set that can be described as follows:

(3) $$W^{\prime }={⋃}_{i=1}^{\frac{n\times 2}{3}}0||w_{i}$$

In Equation (4), W′ can be described as follows:

(4) $$W^{\prime }=\{w^{\prime }:\forall \left|w^{\prime }\right|=4\;;\;\;w^{\prime }=0\|w\}$$

As a result of the operations that prepare the input suitable for the search in the 2D spatial domain, the encoding process can be implemented as explained in the following steps.

Step 4: In this step, each w′ is subjected to an encoding process by considering the arranged 2D spatial domain, as presented in Figure 2a.

The usage of the 2D spatial domain represents a basis for further developments. Note that this coordinate plane has been separated into two regions: Cluster 0 and Cluster 1. In every cluster, the four vectors were chosen as identical in length but different in dimension since there are four DNA bases (A, G, C, T) and four combinations of 2 bits (00, 01, 10, 11). This design is suitable with the traditional bit–base translation presented in Table 2. However, we aimed to increase the encoding efficiency of this traditional scheme. In Figure 2a, notice that there are 8 vectors and that 4 of them are identical. For this reason, cluster regions (Clusters 0 and 1) are necessary. By means of the usage of these clusters in the 2D spatial domain, the 4-bit combination has been increased to 8. When the Cluster 0 region is considered, notice that the LSBs (Least Significant Bits) of C and T are concatenated with 00 and that the LSBs of A and G are concatenated with 01. Similarly, when the Cluster 1 region is considered, notice that the LSBs (Least Significant Bits) of C and T are concatenated with 10 and that the LSBs (Least Significant Bits) of A and G are concatenated with 11. This design enables us to store 3 bits in one DNA base by increasing the traditional 4-bit combinations that match with the four DNA bases to 8. In Figure 2b, these four vectors are provided in detail for ease of understanding. In this study, we aimed to benefit from the vector representations of DNA bases for encoding. Let us call them base vectors.

Let us explain the purpose of the design and usage of the base vector representation and 2D spatial domain using a simple example. Suppose that w = 010 as a result of Steps 1 and 2. It is obvious that w′ is estimated as 0010 after Step 3 by concatenating the MSB of w with 0. When w′ = 0010 is searched in the 2D spatial domain by dividing it into two equal patterns in length, it can be easily observed that it resides in the Cluster 1 region (on the bottom left side in Figure 2a). Moreover, it is obvious that the green base vector matches this w′ pattern, whose value is 0010. Accordingly, it can be estimated that the corresponding base for this w′ pattern is T. Thus, T is obtained for a 010 bit stream whose length is 3.

Let us consider another w = 001 after Steps 1 and 2. Again, after Step 3, w′ is estimated to be 0001 by concatenating the MSB of w with 0. When w′ = 0001 is searched in the 2D spatial domain two by two, it can be seen that this pattern resides in the Cluster 0 region (on the bottom right side in Figure 2a). It is evident that the black base vector matches the w’ pattern, whose value is 0001. Therefore, the corresponding base is G for the w′ pattern. Thus, G is obtained for a 001 bit stream with a length of 3.

For every given binary input, all DNA bases were estimated in this manner. This is the step in which compression occurs. Here, note that three bits are represented by only one DNA base. However, it is very valuable to mention that this encoding exceeds the limitation of traditional DNA base–bit transformation, where two bits are represented as only one DNA base. This result yielded significantly better compression ratios and capacities, which are important performance measures. In Table 3, the computation results including Steps 1–4 have been provided for the given B. Here, B has been separated into words of length three, namely, ws has been estimated (Step 2). Subsequently, the MSB part of every w is concatenated with 0 (Step 3). Thus, we obtain w′. After obtaining w′, we search it on the coordinate plane shown in Figure 2a, two by two. Thus, we obtain the corresponding base vector and cluster given in the 4th and 5th rows of Table 3, respectively.

Step 5: The primer sequence was estimated for the synthesis of the previously obtained DNA sequence in Step 4. This was performed as shown in Table 4.

Consider the last row of Table 3, labelled as cluster. Note that the cluster section is in binary form. Two numbers were used to determine the corresponding primer base for synthesis. This transformation was implemented. For example, for 1 and 0, the first pair corresponds to G (Table 4); for 1 and 0, the second pair corresponds to G. This is the step at which compression ends. Some output instances for Steps 1–5 have been provided in Table 5.

At the end of the encoding, the resulting DNA sequence was formed as follows:

*PRIMER//DNA: GGACGCACGG//TGTAGATTTTTTTGTGTTTA*

**Figure 3:**
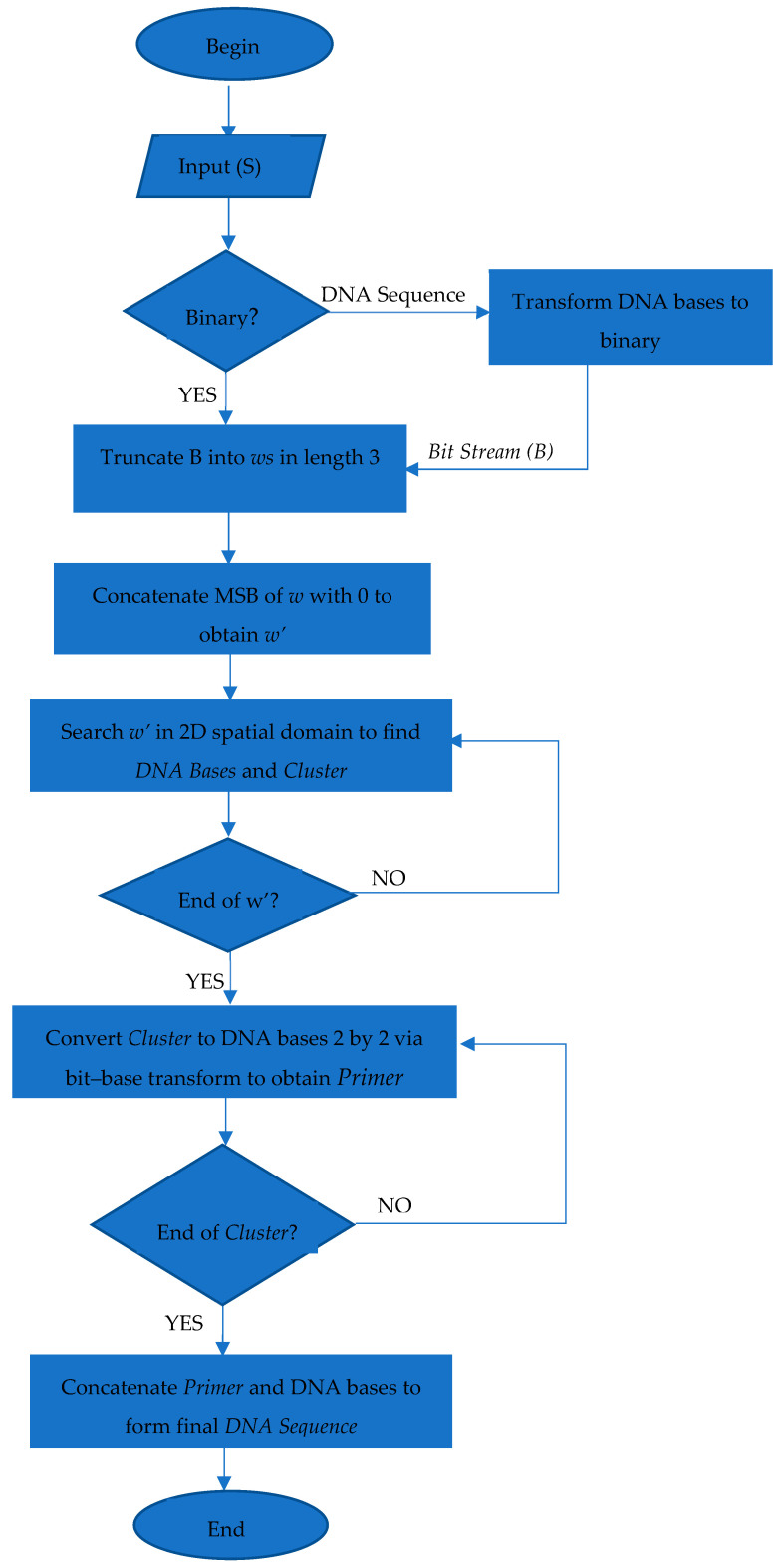
Flowchart of encoding phase.

Here, please note that the Primer sequence is half the length of the DNA Base sequence. This is due to the consideration of numbers in the cluster sequence two by two to find the cluster, as shown in Table 4. A flowchart of the encoding phase is shown in Figure 3.

### 2.3. Decoding Phase of ASCDNA

Step 1. In this step, we begin estimations by considering the output sequence that has been obtained at the end of the encoding phase after Step 5:

*GGACGCACGGTGTAGATTTTTTTGTGTTTA*

Note that 1/3 of the resulting DNA sequence (on the left side) corresponds to primers (refer to the last paragraph of Step 5 in Section 2.2). Accordingly, the sequence can be expressed as

*PRIMER//DNA: GGACGCACGG//TGTAGATTTTTTTGTGTTTA*

Step 2: By considering the primer sequence in Step 1, the correct cluster stream in the form of DNA bases can be obtained as follows:

*GGACGCACGG*

We have to convert this primer sequence to a binary sequence in order to perform reversible estimations on the 2D spatial domain. When Table 4 is considered, it can be observed that G corresponds to 10. This result corresponds to Clusters 1 and 0. The second G value in the sequence corresponds to 10, as shown in Table 4. This result again gives Clusters 1 and 0. Thus, the corresponding cluster stream can be obtained individually in binary form as follows:

*Cluster* = {*1*, *0*, *1*, *0*, *0*, *0*, *1*, *1*, *1*, *0*, *1*, *1*, *0*, *0*, *1*, *1*, *1*, *0*, *1*, *0*}

Step 3: In this step, we have two arguments for obtaining B. We have a cluster stream of binary and DNA bases. Now, it is possible to search for the DNA base of concern in the 2D spatial domain (Figure 2a). Thus far, we have obtained the cluster from Step 2 and DNA base from Step 1 (which is the output of the encoding phase).

*DNA Base* = {*T*, *G*, *T*, *A*, *G*, *A*, *T*, *T*, *T*, *T*, *T*, *T*, *T*, *G*, *T*, *G*, *T*, *T*, *T*, *A*}

*Cluster* = {*1*, *0*, *1*, *0*, *0*, *0*, *1*, *1*, *1*, *0*, *1*, *1*, *0*, *0*, *1*, *1*, *1*, *0*, *1*, *0*}

Let us consider the first elements of each array, T, and 1. This means searching the base T vector in Cluster 1 by employing a 2D spatial domain to obtain w′ (please refer to Figure 2a,b). Accordingly, we obtained the corresponding bit pattern for base T in Cluster 1, which is 0010. Each w′ for each element in the DNA base can be estimated in this manner. Thus, W′ is obtained.

Step 4: In this step, each w ϵ W is obtained by omitting 0 from the MSB of each w′ ϵ W′. Recall the structure of w′ ϵ W′ from Equations (3) and (4)

(3) $$W^{\prime }={⋃}_{i=1}^{\frac{n\times 2}{3}}0||w_{i}$$

(4) $$W^{\prime }=\{w^{\prime }:\forall \left|w^{\prime }\right|=4,\;w^{\prime }=0\left\|w\right\}$$

By omitting 0 from the MSB of w′, we obtain W as

(5) $$W={⋃}_{i=1}^{\frac{n\times 2}{3}}{Truncate\;(MSB,\;\;w}_{i}^{\prime })$$

The words W can be described as follows:

(6) $$W=\{w:\forall \left|w\right|=3\;\&\;w={Truncate\;(MSB,\;\;w}_{i}^{\prime })\}$$

For instance, remember that w′ = 0010 from the previous step. We can obtain w by omitting 0 from the MSB. Thus, w = 010. All ws in W can be obtained in the same way by searching for the next DNA base in the cluster of concern in the 2D spatial domain.

Step 5: After obtaining W, we concatenate its elements to obtain B:

(7) $$B={⋃}_{i=1}^{\frac{n\times 2}{3}}w_{i}||w_{i+1}$$

Please remember that B was formed by a traditional 2-bit per symbol DNA substitution if the input is a DNA sequence. Finally, if necessary, by employing the reverse of this two-by-two substitution procedure, the original input DNA sequence is obtained as follows:

(8) $$S={⋃}_{i=1}^{n\times 2}Reverse\_Substitution\;(b_{i},b_{i+1})$$

**Figure 4:**
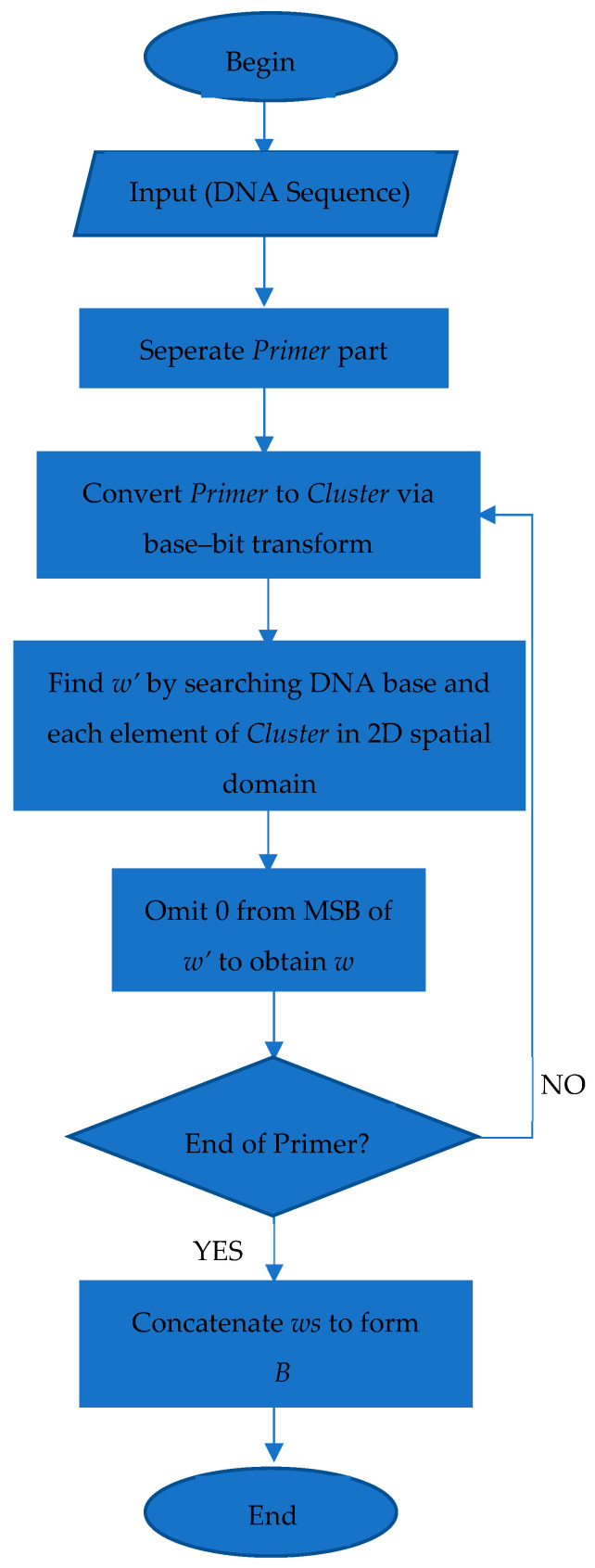
Flowchart of decoding phase.

A flowchart of the decoding phase is shown in Figure 4.

## 3. Experimental Results

In this section, experiments are conducted by providing the storage capacity, compression ratio, GC content, and reliability analyses. Moreover, a comparison of the proposed method with other contemporary methods in the literature was carried out by considering the storage capacity, which is widely used, and a concrete measurement criterion. The experiments were conducted using the C# programming language on a personal computer with an Intel i7 2.2 GHz CPU and 6 GB of RAM.

### 3.1. Storage Capacity

Simulation experiments were performed to measure the storage capacity. The storage capacity is computed in terms of bits stored per nucleotide, *bpn*, which is defined as follows [9]:

(9) $$bpn=\frac{bits\;stored}{sequence\;length}$$

In Equation (9), “the stored bits” indicate the total number of bits stored in DNA. “sequence length” indicates the total number of bases in DNA. For unbiased evaluation, the proposed method was applied to a standard dataset of DNA sequences. The dataset contains 11 sequences, including two chloroplast genomes (CHMPXX and CHNTXX), five human genes (HUMDYSTROP, HUMGHCSA, HUMHBB, HUMHDABCD, and HUMHPRTB), two mitochondrial genomes (MPOMTCG and MTPACG), and two viral genomes (HEHCMVCG and VACCG). The details of the employed dataset files and the results of the experiments are presented in Table 6.

**Figure 5:**
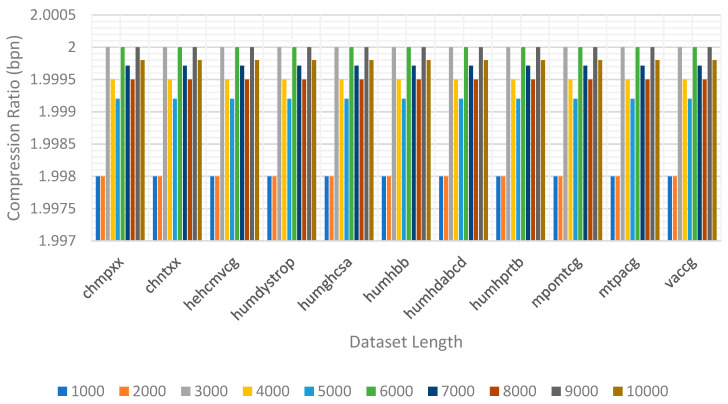
Compression ratios according to dataset types and lengths.

In Table 6, the encoding phase was performed by employing the dataset files provided in the left column. The obtained capacity ratios are presented in terms of bits per nucleotide (bpn). It can be obviously seen that the obtained bpn values stayed stable and that the average bpn value is close to 2. In Figure 5, the bpn rates were investigated by incrementing the dataset length 1000 by 1000 to 10,000 bases. We performed 10 investigations per dataset, for a total of 110 investigations. Each dataset was investigated, and the bpn rates were estimated separately. In Figure 5, it can be seen that the bpn values were approximately 1.99 and stayed stable.

In contrast to the experiments and comparisons mentioned above, the proposed method has been compared with other contemporary methods in the literature by considering the capacity in terms of bpn, as presented in Table 7. The reason for considering capacity as a comparison criterion is its wide usage and the fact that it is a concrete and reachable content in many papers. The carrier medium and storage capacities in each study are presented in Table 7. The storage capacity was increased to 1.99 by the proposed method.

### 3.2. Compression Ratio

The compression ratio (CR), which is another widely used performance metric in many compression techniques, is computed according to the following formula:

(10) $$CR(\%)=\frac{S_{out}}{S_{in}}\times 100$$

where S_out_ is the compressed stream (after compression) and S_in_ is the input stream size (before compression). The lower the CR value is, the more efficient the compression [30]. As can be seen from Table 8, the CR stayed stable at 50%.

In order to have a concrete example, let us consider the input stream given in Table 5 (please refer to Section 2.2.):

*B* (*Bit Stream*) *=* { 010 001 010 101 001 101 010 010 010 000 010 010 000 001 010 011 010 000 010 101}

Here, we have 60 symbols in this input stream. Then, let us consider the resultant stream, which is the concatenated form of the primer and DNA bases:

*PRIMER//DNA*: {*GGACGCACGGTGTAGATTTTTTTGTGTTTA*}

There are 30 symbols in the output stream. It is obvious that a 50% CR was obtained when the symbols in both the input and output streams were rated.

### 3.3. GC Content

As previously mentioned, the four basic bases of the DNA sequence are A, T, C, and G. In DNA, the *GC* content determines the thermal stability of the sequence [28]. For DNA sequences with length *n*, the *GC* content is computed as follows:

(11) $${GC}_{n}=\frac{\left|G\right|+|C|}{\left|n\right|}$$

The smaller the ratio, the more stable are the physical properties of the sequences [31]. The details of the experiments performed for the GC content are provided in Table 9.

For the proposed method, the average GC content was estimated as 46.2%, as shown in Table 9. Because the GC content affects the DNA data storage stability and read accuracy, this rate is sufficiently high to ensure the recovery of the original input data [8].

### 3.4. Reliability

**Figure 6:**
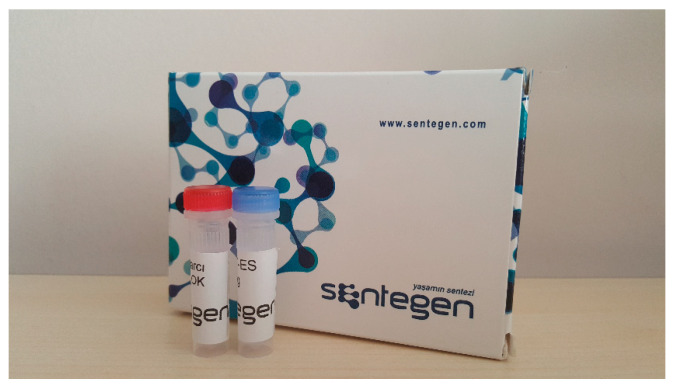
The synthesized DNA chain for conservation.

In the proposed study, a 9588 bp chain of DNA was synthesized with the support of Düzce University Scientific Research Projects Coordinators. The synthesized DNA chains were stored in tubes as shown in Figure 6.

**Figure 7:**
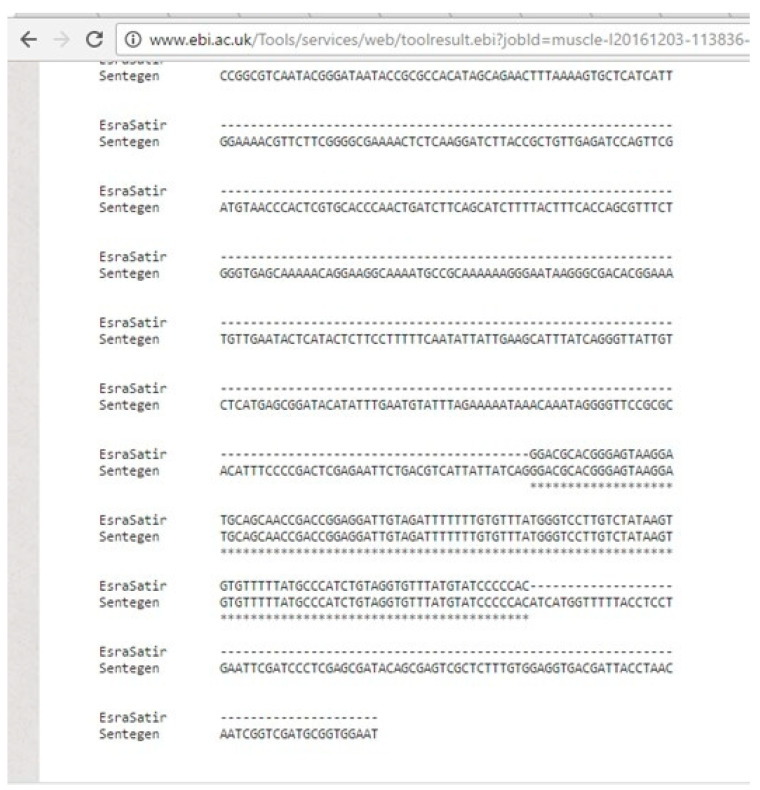
The detailed multiple reading results for the same input.

After synthesizing the DNA chain, it is essential to obtain the original DNA sequence by re-reading it. This process is known as DNA sequencing. The details of the multiple sequence analysis for the same input are presented in Figure 7. The experiments showed that the original input stream could be obtained without any information loss. Here, after sequencing the 9588 bp DNA chain, it was observed that the synthesized and sequenced DNA chains matched with each other without any loss of information. This synthesized 9588 bp DNA chain was encapsulated in a plasmid for ease of storage.

## 4. Discussion

In recent years, significant efforts have been made to adapt digital data storage processes to computational biology tasks. With the development of Sanger sequencing technology, DNA sequences have been collected and manipulated using computers. This has yielded a new and attractive field of bioinformatics and DNA data storage.

In this study, a DNA data storage method using spatial encoding-based lossless compression, which is based on the vector representation of DNA bases and processing them on a 2D spatial domain, was proposed. It was observed that the experimental results of the proposed method had better accuracy rates than those of previously known algorithms in the literature when the storage capacity is considered in terms of bpn. Moreover, significant compression ratios for storage and GC content values for the stability of the sequence were achieved. The reliability of the proposed method was evaluated by performing multiple DNA sequencing analyses. The proposed method has a storage capacity of 1.99 in terms of bpn. This was mainly due to the use of the 2D spatial domain and vector representation of DNA bases in the proposed study. Another advantage of the proposed approach is that it enables the storage of 3 bits in one DNA base. This exceeds traditional encoding schemes, where 2 bits are stored in one DNA base. In addition, this method occupies a small area of memory during the simulation.

However, increasing the storage capacity via the compression ratio is a valuable problem that needs to be addressed. In the proposed method, 3 bits correspond to one base. First, we aim to increase this rate in future studies. We aim to address this issue by adapting the proposed method to the 3D spatial domain. In this way, we aim to benefit from the variations in the directions of vector representations to increase the amount of stored bits in one DNA base. In addition, this will enable us to use error-correcting codes (if needed) without affecting the storage capacity. Another important issue that needs to be addressed is the practical running time. DNA will not be fast enough to compete with optical, magnetic, or quantum formats in the foreseeable future. The use of primers renders the DNA strand accessible during sequencing. In addition, to provide a more developed random-access mechanism, hash codes can be used as fingerprints. Finally, the implementation of the proposed method using living organisms and mutation resistance must be investigated. This presents a new challenge by enabling the proposed method to carry information from one generation to another. However, this process requires a long, detailed, and expensive in vivo experiment because the preservation of the synthesized DNA is difficult in living organisms owing to mutations and amplifications.

## Figures and Tables

**Table 1:** Digital information carriers [4].

Carrier Medium	Carrier Capacity	Time to Access	Service Life
SSD (Solid State Drive)	≈1 TB	ms	≈10 years
CD, DVD, Blue Ray disks	≈128 GB	ms	≈tens of years
HDD (Hard Disk Drive)	≈10 TB	10 ms	≈10 years
Magnetic Tapes	≈100 TB	minutes	≈15–30 years
DNA	≈hundreds of EB	tens of hours	centuries

**Table 2:** Bit–base translation table [9].

Bits in Computer Systems	Oligos in DNA Molecule
00	T
01	G
10	C
11	A

**Table 3:** Sample computation results for Steps 1–4.

B (Bit Stream)	010001010101001101010010010000010010000001010011010000010101																			
*w*	010 001 010 101 001 101 010 010 010 000 010 010 000 001 010 011 010 000 010 101																			
*w*′	0010 0001 0010 0101 0001 0101 0010 0010 0010 0000 0010 0010 0000 0001 0010 0011 0010 0000 0010 0101																			
DNA Base	T	G	T	A	G	A	T	T	T	T	T	T	T	G	T	G	T	T	T	A
Cluster	1	0	1	0	0	0	1	1	1	0	1	1	0	0	1	1	1	0	1	0

**Table 4:** Base–bit translation table.

A	00
T	01
G	10
C	11

**Table 5:** Computation results for Steps 1–5.

B (Bit Stream)	010 001 010 101 001 101 010 010 010 000 010 010 000 001 010 011 010 000 010 101																			
*w*	010 001 010 101 001 101 010 010 010 000 010 010 000 001 010 011 010 000 010 101																			
*w*′	0010 0001 0010 0101 0001 0101 0010 0010 0010 0000 0010 0010 0000 0001 0010 0011 0010 0000 0010 0101																			
DNA Base	T	G	T	A	G	A	T	T	T	T	T	T	T	G	T	G	T	T	T	A
Cluster	1	0	1	0	0	0	1	1	1	0	1	1	0	0	1	1	1	0	1	0
Primer	G		G		A		C		G		C		A		C		G		G	

**Table 6:** Experimental results in terms of bpn according to datasets.

Sequence	Bits Before Compression (Base × 8)	Base After Compression	Capacity (Bits/Bases-bpn)
CHMPXX	968,192	484,096	1.99999587
CHNTXX	1,246,752	623,376	2
HEHCMVCG	1,834,832	917,416	1.99999782
HUMDYSTROP	310,160	155,080	1.9999871
HUMGHCSA	531,960	265,980	2
HUMHBB	586,464	293,232	2
HUMHDAB	470,912	235,456	1.99999151
HUMHPRTB	453,896	226,948	1.99999119
MPOMTCG	1,492,864	746,432	1.99999866
MTPACG	802,512	401,256	2
-VACCG	1,533,896	766,948	1.99999739
Average			1.999996322

**Table 7:** Comparison results of the proposed method with other contemporary methods [4].

Reference	Carrier	Short Description	Capacity (bpn)
Kac and Ascott (2000) [17]	*E.coli* plasmid	They created a gene by translating a sentence from the biblical book of Genesis into Morse Code and converting the Morse Code into DNA bases via a special conversion principle.	1.75
Church et al. (2012) [18]	synthetic	They developed a strategy where a 5.27-megabit book was written using DNA microchips and read the book by using next-generation DNA sequencing.	0.6
Goldman et al. (2013) [19]	synthetic	They encoded 739 kilobytes of hard-disk storage in DNA and synthesized this DNA. Then, they sequenced it and reconstructed the original files with 100% accuracy.	0.33
Hafeez et al. (2014) [9]	Amine acids	Mentioned in Section 1.	0.52
Grass et al. (2015) [20]	synthetic	They encapsulated DNA in an inorganic matrix and employed error-correcting codes for storage. They translated 83 kB of information to 4991 DNA segments that were encapsulated in silica.	1.187
Yazdi et al. (2015) [21]	synthetic	They described the first DNA-based storage architecture that enables random access to data blocks and the rewriting of information stored at arbitrary locations within the blocks.	1.575
Blawat et al. (2016) [22]	synthetic	They developed an efficient forward error-correcting scheme adapted to DNA channels. They stored and retrieved error-free 22 MByte of digital data in synthetic DNA.	0.89
Yazdi et al. (2017) [23]	synthetic	They designed a portable, random-access platform that may be implemented in practice using nanopore sequencers.	1.72
Erlich and Zielinski (2017) [24]	synthetic	They presented the DNA Fountain method that is highly robust and approaches the information capacity per nucleotide. They stored a total of 2.14 × 106 bytes in DNA and retrieved the information successfully.	1.55
Organick et al. (2018) [25]	synthetic	They stored 35 different files from 29 Kb to 44 Mb including the “Declaration of Human Rights” in more than 100 languages, music video “This Too Shall Pass”, and a list of plant species from the seed database stored at the Global Seed Vault in Svalbard.	0.81
Lee et al. (2018) [10]	synthetic	Mentioned in Section 1.	≈0.4
Wang et al. (2019) [26]	synthetic	They devised a DNA data storage scheme with variable-length oligonucleotides, where a hybrid DNA mapping scheme converts digital data to DNA records.	1.67
Antkowiak et al. (2020) [27]	synthetic	They presented a DNA storage system that relies on massively parallel light-directed synthesis, which is considerably cheaper than conventional solid-phase synthesis.	0.94
Yang et al. (2020) [28]	synthetic	They described a new molecular chemotype for data archiving based on the unnatural genetic framework of α-l-threofuranosyl nucleic acid (TNA).	1.0
Chen et al. (2021) [29]	Organic *	They presented the de novo design and synthesis of an artificial chromosome that encodes two pictures and a video clip.	1.19
Lenz and Zeh (2023) [12]	synthetic	Mentioned in Section 1.	1.0
Hong et al. (2024) [8]	synthetic	Mentioned in Section 1.	1.75
PELMİ (2024) [14]	synthetic	Mentioned in Section 1.	1.48
The proposed method	synthetic	Explained in Section 2.	1.99

*: Artificial yeast chromosome.

**Table 8:** Compression ratio results according to datasets.

Sequence	Bits Before Compression (Base × 8)	Bases After Compression	Compression Ratio (%)
CHMPXX	968,192	484,096	50
CHNTXX	1,246,752	623,376	50
HEHCMVCG	1,834,832	917,416	50
HUMDYSTROP	310,160	155,080	50
HUMGHCSA	531,960	265,980	50
HUMHBB	586,464	293,232	50
HUMHDAB	470,912	235,456	50
HUMHPRTB	453,896	226,948	50
MPOMTCG	1,492,864	746,432	50
MTPACG	802,512	401,256	50
VACCG	1,533,896	766,948	50
Average			50

**Table 9:** GC content results according to oligo length.

Length of Oligos	Frequency of Oligos	GC Content
100	A = 21	45
	G = 29	
	C = 16	
	T = 34	
1000	A = 120	535
	G = 235	
	C = 300	
	T = 345	
10,000	A = 2504	5243
	G = 2487	
	C = 2756	
	T = 2253	
100,000	A = 32,155	34,110
	G = 17,569	
	C = 16,541	
	T = 33,735	

## Data Availability

The following 11 benchmark standard datasets of DNA sequences were used in this study. The datasets consist of two chloroplast genomes (CHMPXX and CHNTXX), five human genes (HUMDYSTROP, HUMGHCSA, HUMHDABCD, HUMHBB, and HUMHPRTB), two mitochondria genomes (MPOMTCG and MTPACG); two viral genomes (HEHCMVCG and VACCG). The data used in the scope of the experiments performed in this study can be accessed via the provided link: https://encode.su/threads/2105-DNA-Corpus (accessed on 19 December 2024).

## References

[1] Lin Y., Xie Z., Chen T., Cheng X., Wen H. (2024). Image privacy protection scheme based on high-quality reconstruction DCT compression and nonlinear dynamics. Expert. Syst. Appl..

[2] Li H., Zhang L., Cao H., Wu Y. (2023). Hash Based DNA Computing Algorithm for Image Encryption. Appl. Sci..

[3] Song L., Geng F., Gong Z.Y., Chen X., Tang J., Gong C., Zhou L., Xia R., Han M.Z., Xu J.Y. (2022). Robust data storage in DNA by de Bruijn graph-based de novo strand assembly. Nat. Commun..

[4] Garafutdinov R.R., Chemeris D.A., Sakhabutdinova A.R., Kiryanova O.Y., Mikhaylenko C.I., Chemeris A.V. (2022). Chemeris, Encoding of non-biological information for its long-term storage in DNA. Biosystems.

[5] Lee S.-J., Cho G.-Y., Ikeno F., Lee T.-R. (2018). BAQALC: Blockchain Applied Lossless Efficient Transmission of DNA Sequencing Data for Next Generation Medical Informatics. Appl. Sci..

[6] Tong J., Han G., Sun Y. (2023). An Improved Marker Code Scheme Based on Nucleotide Bases for DNA Data Storage. Appl. Sci..

[7] Zhirnov V., Zadegan R.M., Sandhu G.S., Church G.M., Hughes W.L. (2016). Nucleic acid memory. Nat. Mater..

[8] Hong J., Rasool A., Wang S., Ziou D., Jiang Q. (2024). VSD: A Novel Method for Video Segmentation and Storage in DNA Using RS Code. Mathematics.

[9] Hafeez I., Khan A., Qadir A. (2014). DNA-LCEB: A high-capacity and mutation-resistant DNA data-hiding approach by employing encryption, error correcting codes, and hybrid twofold and fourfold codon-based strategy for synonymous substitution in amino acids. Med. Biol. Eng. Comput..

[10] Lee S.H., Lee E.J., Hwang W.J., Kwon K.R. (2018). Reversible DNA data hiding using multiple difference expansions for DNA authentication and storage. Multimed. Tools Appl..

[11] Rahman M.S., Khalil I., Yi X. (2019). A lossless DNA data hiding approach for data authenticity in mobile cloud based healthcare systems. Int. J. Inf. Manag..

[12] Lenz A., Siegel P.H., Wachter-Zeh A., Yaakobi E. (2023). The Noisy Drawing Channel: Reliable Data Storage in DNA Sequences. IEEE Trans. Inf. Theory.

[13] Preuss I., Rosenberg M., Yakhini Z., Anavy L. (2024). Efficient DNA-based data storage using shortmer combinatorial encoding. Sci. Rep..

[14] Cao B., Wang K., Xie L., Zhang J., Zhao Y., Wang B., Zheng P. (2024). PELMI: Realize robust DNA image storage under general errors via parity encoding and local mean iteration. Brief. Bioinform..

[15] Sukumaran S.C., Mohammed M. (2021). PCR and Bio-signature for data confidentiality and integrity in mobile cloud computing. J. King Saud. Univ. Comput. Inf. Sci..

[16] Cao M.D., Dix T.I., Allison L., Mears C. A Simple Statistical Algorithm for Biological Sequence Compression. Proceedings of the 2007 Data Compression Conference (DCC’07).

[17] Kac E., Ascott R. (2000). Genesis: A Transgenic Artwork. Art, Technology, Consciousness: Mind@large.

[18] Church G.M., Gao Y., Kosuri S. (2012). Next-generation digital information storage in DNA. Science.

[19] Goldman N., Bertone P., Chen S., Dessimoz C., LeProust E.M., Sipos B., Birney E. (2013). Towards practical, high-capacity, low-maintenance information storage in synthesized DNA. Nature.

[20] Grass R.N., Heckel R., Puddu M., Paunescu D., Stark W.J. (2015). Robust chemical preservation of digital information on DNA in silica with error-correcting codes. Angew. Chem. Int. Ed. Engl..

[21] Yazdi S.M.H.T., Yuan Y., Ma J., Zhao H., Milenkovic O. (2015). A rewritable, random-access DNA-based storage system. Sci. Rep..

[22] Blawat M., Gaedke K., Huetter I., Chen X.-M., Turczyk B., Inverso S., Pruitt B., Church G. (2016). Forward error correction for DNA data storage. Procedia Comput. Sci..

[23] Yazdi S.M.H.T., Gabrys R., Milenkovic O. (2017). Portable and error-free DNA-based data storage. Sci. Rep..

[24] Erlich Y., Zielinski D. (2017). DNA Fountain enables a robust and efficient storage architecture. Science.

[25] Organick L., Ang S.D., Chen Y.J., Lopez R., Yekhanin S., Makarychev K., Racz M.Z., Kamath G., Gopalan P., Nguyen B. (2018). Random access in large-scale DNA data storage. Nat. Biotechnol..

[26] Wang Y., Noor-A-Rahim M., Zhang J., Gunawan E., Guan Y.L., Poh C.L. (2019). High capacity DNA data storage with variable-length oligonucleotides using repeat accumulate code and hybrid mapping. J. Biol. Eng..

[27] Antkowiak P.L., Lietard J., Darestani M.Z., Somoza M.M., Stark W.J., Heckel R., Grass R.N. (2020). Low cost DNA data storage using photolithographic synthesis and advanced information reconstruction and error correction. Nat. Commun..

[28] Yang K., McCloskey C.M., Chaput J.C. (2020). Reading and writing digital information in TNA. ACS Synth. Biol..

[29] Chen W., Han M., Zhou J., Ge Q., Wang P., Zhang X., Zhu S., Song L., Yuan Y. (2021). An artificial chromosome for data storage. Natl. Sci. Rev..

[30] Chłopkowski M., Walkowiak R. (2015). A general purpose lossless data compression method for GPU. J. Parallel Distrib. Comput..

[31] Li X., Zhou S., Zou L. (2022). Design of DNA Storage Coding with Enhanced Constraints. Entropy.

